# Prognostic significance of tumour stroma ratio in inflammatory breast cancer

**DOI:** 10.1186/s40064-015-0852-7

**Published:** 2015-02-10

**Authors:** Candice L Downey, Helene H Thygesen, Nisha Sharma, Abeer M Shaaban

**Affiliations:** St James’s University Hospital, Leeds, LS9 7TF UK; Leeds Institute of Cancer & Pathology, University of Leeds, St James’s University Hospital, Leeds, LS9 7TF UK; Department of Cellular Pathology, Queen Elizabeth Hospital Birmingham and University of Birmingham, Birmingham, B15 2TW UK

**Keywords:** Breast cancer, Inflammatory, Stroma, Tumour-stroma ratio, Prognosis

## Abstract

Tumour stroma ratio (TSR) is emerging as an important prognostic indicator in cancer. We have previously shown TSR to be prognostic in oestrogen receptor positive breast cancer. Its role in inflammatory breast cancer, a rare but aggressive form of breast cancer, has not been identified. Here we aimed to determine the prognostic significance of TSR in a cohort of patients with inflammatory breast carcinoma.

TSR was measured by point counting virtual H&E stained tissue sections in 45 inflammatory breast cancer cases. The whole tumour area was sampled. Optimum cut-offs to distinguish high and low TSR was determined by log-rank test. The relationship of TSR to overall survival and disease-free survival (DFS) was analysed alongside multivariate analysis.

The optimal cut-offs between high and low TSR were determined to be 31% for OS and 46% for DFS. There was no significant difference in OS (p = 0.53) nor DFS (p = 0.66) between high and low TSR groups. Multivariate analysis did not demonstrate any new trends, within the limits of a small data sample. A significant correlation was found between pathological response to neoadjuvant chemotherapy and survival (p = 0.008).

There is no evidence that TSR has prognostic significance in inflammatory breast cancer. When compared with published data in non-inflammatory breast carcinoma, this supports the view that differences in stromal biology exist between tumour types and highlights the importance of considering this when interpreting the prognostic value of TSR. However, these findings must be interpreted in the light of the small sample size.

## Purpose

Tumour-stromal ratio (TSR) is fast emerging as a significant prognostic indicator in cancer. The importance of TSR has been investigated in cancers including prostate (Yanagisawa et al. 2007), colorectal (West et al. 2010), oesophageal (Wang et al. 2012) and breast (de Kruijf et al. 2011) although results so far have been inconsistent.

Breast cancer is the most common cancer in the United Kingdom and the second most common cause of death from cancer in women after lung cancer (Office for National Statistics 2013). The vast heterogeneity of the disease necessitates multiple treatment options. Inflammatory breast cancer (IBC) is diagnosed clinically and it is characterized by rapid onset of diffuse erythema and warmth and oedema of the skin of the breast (peau d’orange). It is thought that emboli in lymphatic vessels cause their obstruction and prevents proper drainage of the lymph fluid thus causing swelling of the breast and its inflammatory-like appearance (Robertson et al. 2010). In 2011, an international panel published consensus criteria for the diagnosis of IBC (Dawood et al. 2011). More recently, a UK perspective on the diagnostic criteria, management, documentation and research directions has been proposed (Rea et al., Br J Cancer, in press).

Previous studies into TSR in breast cancer have largely focussed on hormone status, particularly triple-negative; that is, negative for ER, PR and HER2. Moorman et al. showed that a high percentage of stroma predicts poor survival in triple-negative breast cancers (Moorman et al. 2012). TSR was found to be an independent prognostic factor for relapse-free survival in breast cancer patients, and especially in those with triple-negative disease (de Kruijf et al. 2011). However, a study of unselected breast cancer cases showed that TSR did not predict survival on multivariate analysis (Ahn et al. 2012). In addition, our previous study of ER-positive breast cancers demonstrated that a high TSR was related to worse survival across both genders (Downey et al. 2014). The prognostic role of TSR in inflammatory breast carcinoma has not been previously studied.

Here we aim to determine the prognostic significance of TSR in inflammatory breast cancer. The results will help to elucidate the scope of significance of TSR in breast cancer.

## Methods

### Ethical approval

Ethical approval for the study was granted by Leeds (East) Research Ethics Committee (Reference no: 06/Q1206/180).

### Ethical standards

All the work detailed herein was performed to comply with the current laws of the United Kingdom.

### Patients

Patients with the diagnosis of inflammatory breast cancer between the period 2005–2013 were identified from the clinical database at the Leeds Teaching Hospitals NHS Trust. H&E slides and paraffin-embedded tumour blocks of the original (pre-treatment) core biopsy were retrieved from the pathology archives. Comprehensive histological and clinical data including response to chemotherapy (complete, partial, minimal or no response), disease-free survival and overall survival were collected on those patients.

### Clinicopathological data

Histopathological data was obtained from pathology databases. Relapse-free survival and overall survival was available for each patient.

### Measurement of stromal density

Four μm thick haematoxylin and eosin-stained tissue sections were prepared according to standard protocols. Each slide was scanned at 20× magnification with an automated scanning system (Aperio XT, Aperio Technologies, Vista, CA, USA). Using a digital slide viewer (ImageScope version 8.0, Aperio Technologies), the whole area was selected for analysis. A grid with a systematic random sample of 300 points was then superimposed on the selected area using virtual graticule software (Treanor et al. 2008). The number of measurement points was consistent with that found accurate by previous studies (West et al. 2010). The histopathological category under each point was recorded and the number of points attributable to each category was counted. Categories used were tumour, stroma and non-informative (unclassifiable). Points falling on areas of lumen, necrosis, blood vessels, inflammation or blank areas fell into the latter category. TSR was expressed as a percentage of all the informative points per section.

### Statistical analyses

Statistical analysis was performed using GraphPad Prism version 6, R version 2.15 and the Survival package for R. The optimal cut-off value for TSR was calculated as follows: for each tumour proportion-value occurring in the data set, a log-rank test was performed based on a comparison of the group of patients with a TSR ≤ that value, and the patients with P above that. Primary endpoints were disease-free survival (RFS) and overall survival. Univariate survival analyses were performed using Kaplan-Meier curves. Differences between the groups were assessed using the log-rank test. P-values of < 0.05 were considered to be statistically significant. Multivariate analysis was performed using Cox’s Proportional Hazards model.

## Results

A total of 45 patients with available tumour slides/blocks were identified. The mean age at diagnosis was 55 years (+/− 12.8 years, range 32–84 years).

Histopathological data obtained from pathology databases is summarised in Table 1. The majority of the tumours were of ductal no special type, node positive, hormone receptor and HER2 negative.

**Table 1:** **Clinicopathological details of cohort**

**Characteristic**	**(total = 45)**
Mean age (range)	55 (32–84)
Type	
Ductal	33 (73%)
Lobular	7 (16%)
Other	5 (11%)
Grade	
1	3 (7%)
2	20 (44%)
3	22 (49%)
LN	
N0	9 (20%)
N1-3	36 (80%)
ER	
+	20 (44%)
-	25 (56%)
PR	
+	11 (24%)
-	34 (76%)
HER2	
+	13 (29%)
-	32 (71%)
Size	
≤20 mm	14 (31%)
>20 mm	29 (64%)
n/a	2 (4%)

In order to determine optimum cut-offs to distinguish high and low TSR, log-rank tests were performed to compare groups of patients with TSR below or equal to that value, with patients with TSR above that. The cut-off values that led to the smallest p-value were 0.31 for OS and 0.46 for DFS.

Median overall survival was 75 months in the high TSR group and 35 months in the low TSR group. A similar pattern was seen with the disease-free survival: 57.5 months in the high TSR group compared to 37 months in the low TSR group (Table 2).

**Table 2:** **Median OS and DFS between TSR groups**

**Median survival**	**High TSR**	**Low TSR**
OS	75	35
DFS	57.5	37

**Figure 1:**
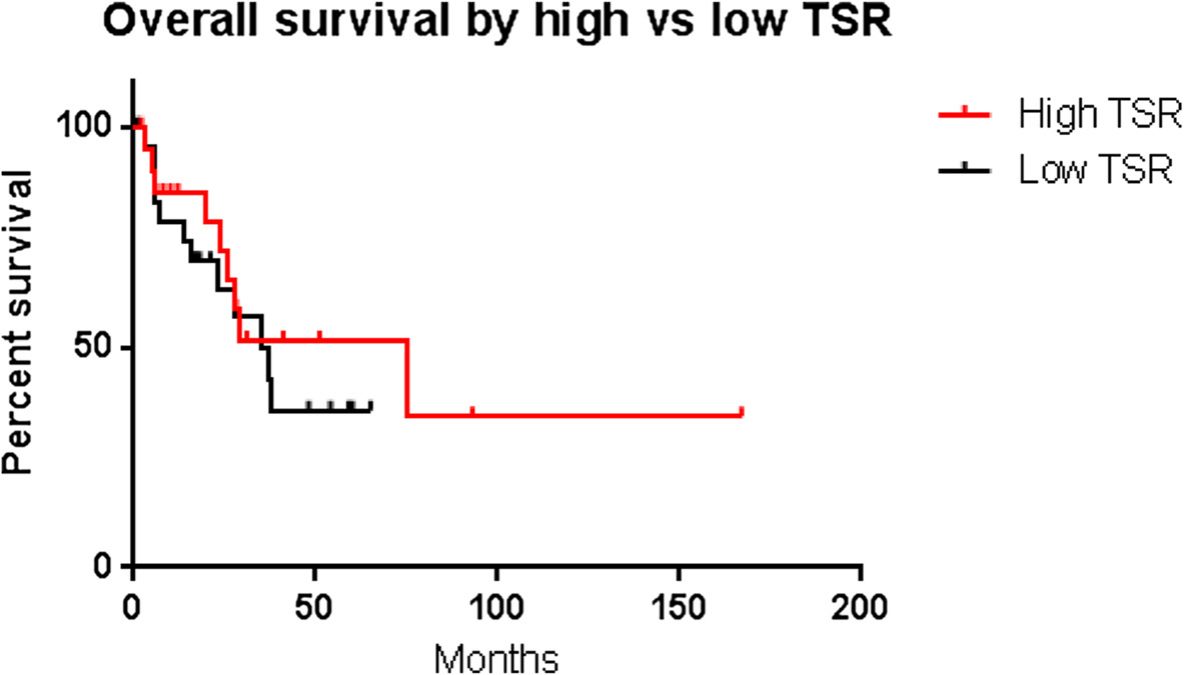
**Kaplan-Meier survival curves showing overall survival after stratification by TSR.**

**Figure 2:**
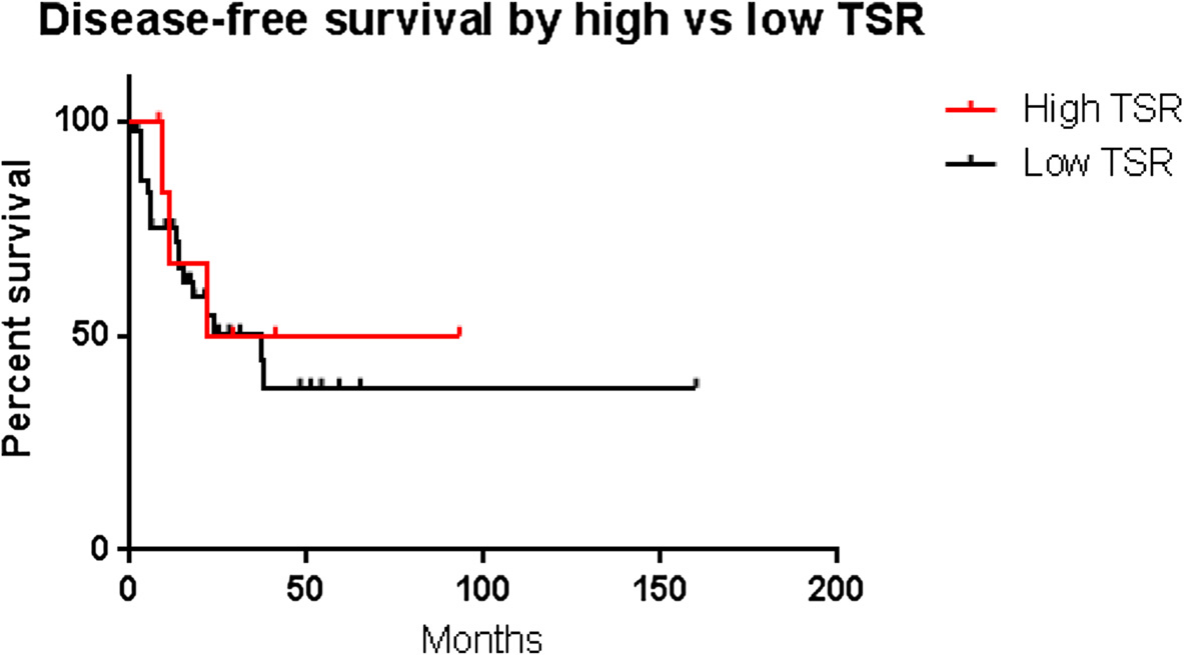
**Kaplan-Meier survival curves showing disease-free survival after stratification by TSR.**

There was no significant difference in survival between high and low TSR groups. This was true for both OS (p = 0.53, Figure 1) and DFS (p = 0.66, Figure 2).

Multivariate analysis demonstrated that pathological response (minimal or no response versus partial/complete response) correlated with survival (p = 0.008) but TSR was not an independent prognostic factor for survival.

## Discussion

The role of stromal microenvironment in dictating tumour behaviour has recently been emphasised. The initiation, growth and progression of cancer is dependent on the tumour microenvironment of which tumour stroma is an integral part. Recently attention has focused on the potential prognostic value of TSR in different types of cancer. In general, low TSR appears to be associated with worse prognosis; in breast (de Kruijf et al. 2011); (Moorman et al. 2012); (Ahn et al. 2012); lung (Maeshima et al. 2002); prostate (Yanagisawa et al. 2007); stomach (Wu et al. 2013)and colon and rectum (West et al. 2010); (Mesker et al. 2007).

Current literature describes conflicting results, especially in breast, and seemingly dependent on hormone status. Whereas a number of studies on triple-negative breast cancers have found TSR to predict poor survival (de Kruijf et al. 2011; Moorman et al. 2012; Dekker et al. 2013), this prognostic value is diminished in studies of unselected breast cancers (Ahn et al. 2012). A study of ER-positive breast cancers demonstrated that a high TSR was related to worse survival across both genders (Downey et al. 2014) suggesting that the importance of TSR in breast cancer may be dependent on key molecular determinants of tumour subtype.

This is the first study to analyse stromal density and its relation to prognosis in inflammatory breast cancer. Our findings provide no evidence of any prognostic significance of TSR in inflammatory breast cancer. This finding adds to the literature base.

There are a number of methodological issues in this study. While there was a difference in survival between high and low TSR (75 months versus 35 months) suggestive of better outcome in the high stromal group, this did not reach statistical significance. Although 45 cases represent a relatively large cohort in this rare disease, the number of cases is small and the study is likely to be underpowered. The results need to be interpreted in this context.

Our findings suggest that differences in stromal biology may exist between tumour subtypes and highlights the importance of tumour subtype when interpreting the prognostic value of TSR. Future studies may elucidate crucial stromal differences between tumour subtypes which could better predict survival.
